# “It’s Allowed Me to Be a Lot Kinder to Myself”: Exploration of the Self-Transformative Properties of Solitude During COVID-19 Lockdowns

**DOI:** 10.1177/00221678231157796

**Published:** 2023-03-06

**Authors:** Jodie Paterson, Miriam Sang-Ah Park

**Affiliations:** 1 Nottingham Trent University, UK

**Keywords:** solitude, authenticity, COVID-19, self-concept, psychological well-being

## Abstract

The Coronavirus (COVID-19) national lockdowns profoundly affected the lives of many, as significant portions of the U.K. population were involuntarily sequestered away from their usual social landscapes into newfound states of solitude. Many millennials (those between the ages of 25 and 40 at the point of study), having lived in an age of constant connection, found themselves in an extended period of solitude for the first time. The current qualitative study explores through Interpretative Phenomenological Analysis (IPA) how some UK millennials were able to harness the unique self-transformative properties of positive solitude during the COVID-19 national lockdowns. Analysis revealed a narrative of self-discovery, as domains of positive solitude granted the participants freedom from the demands of performative social burdens and encouraged opportunities to engage with mindfulness and meaningful introspection. These activities, within the experience of solitude, encouraged an alignment of inward beliefs and outward behaviors for the participants, thus helping them to cultivate a more congruent self-concept and subsequently a heightened sense of authenticity and enhanced psychological well-being. Clinical implications leading on from the current study highlight the importance of mindfulness and other solitude-promoting interventions as a method to ameliorate depressive symptoms and improve psychological well-being.

## COVID-19, Lockdowns, and Solitude

The Coronavirus (COVID-19), emerging in Hubei province in China in December 2019, reached U.K. shores toward the end of January 2020 (Sohrabi et al., 2020). The World Health Organization (WHO) declared the outbreak as a global pandemic on March 11, 2020, and as of March 23, 2020, the U.K. government introduced strict, mitigating measures in an attempt to stem the spread of the highly infectious virus (Shah & Farrow, 2020). A stringent, national lockdown was imposed upon the U.K. public suddenly, and dramatically changing the shape of the social landscape and the lives of many, sequestering much of the population into new states of solitude and isolation. While imperative for the protection of public health, the uncompromising measure of the national lockdown posed significant risks to mental health and psychological well-being.

It is apparent that the detrimental psychological effects of the lockdowns can be fiercely profound and long lasting (Holmes et al., 2020). The imposed solitude and isolation has directly contributed to confinement-related stress due to social disruptions, increased caregiving burdens, and financial insecurity resulting from job loss and employment vulnerability (Prime et al., 2020). A substantial increase in common mental health problems such as boredom, fear, stress, insomnia, anxiety, and depression (Brooks et al., 2020; Duan & Zhu, 2020; Lima et al., 2020; Pieh et al., 2021) has been observed and experienced, with an additional documented increase in post-traumatic stress disorder (PTSD) diagnosis (Brooks et al., 2020), as well as self-harm and suicidal ideation (Iob et al., 2020; O’Connor et al., 2021). Pierce et al.’s (2020) longitudinal study revealed that lockdown-related deterioration of mental health was observed as early as April 2020 within the U.K. population, and the resulting negative sequelae of the national lockdowns seem undeniable.

Conversely, there is also emerging evidence that the opportunity for solitude, within the context of the COVID-19 lockdowns, has potentially, for some, had a positive impact on mental health and well-being. For example, it has been found that introverted individuals, who show a propensity for solitude, somewhat thrived throughout the imposed lockdowns. Termed as being “emotionally stable lonely,” they expressed a lower level of loneliness and subsequently experienced better mental health (Michinov & Michinov, 2021, p. 9). Time spent in solitude was also found to foster creativity, with research showing that personal levels of creativity flourished during the lockdown resulting in a bolstered resilience against the potential risks of prolonged isolation (Mercier et al., 2021). While there is a paucity of research into potential positive outcomes of the COVID-19 lockdowns, it is imperative that the existence of such experiences are addressed in earnest. For instance, it would be useful to have a broader knowledge base of the outcomes of time spent in solitude on mental health and psychological well-being as such knowledge can lead to initiatives that can improve the quality of life for many. Furthermore, through acknowledgment and validation of variant experiences, a more holistic and thorough understanding of the impacts of the pandemic and the lockdowns can be drawn.

## Solitude and Self-Exploration in Young Adults

Solitude is a discernibly difficult to define phenomenon, with much scholarly debate surrounding the specific parameters of its conceptualization. Some regard solitude as an objective state of being alone, with a strict absence of all aspects of social interaction (Burger, 1995; R. W. Larson, 1990), while others consider it as a state of being alone with oneself, either with or without the presence of others (Long & Averill, 2003; Pauly et al., 2018). Regardless of its definition with respect to physical proximity, what is imperative to the understanding of solitude is the distinct conceptual differentiation it has to the constructs of social isolation and loneliness, with which it can often erroneously be conflated. Succinctly put, isolation and loneliness are both decidedly negative in nature, while solitude is a more multifaceted construct encompassing experiences that are both positive and negative (Pauly et al., 2018).

Solitude can be experienced negatively. Outcomes of solitude, as expected, can be associated with increased loneliness, negative affect, and also maladaptive rumination (Lay et al., 2019). Contextualizing this within a specific setting, for example, those placed within solitary confinement while serving a custodial sentence, individuals have been shown to experience considerable psychological harm including stress-related disorders and increased instances of self-harm and PTSD (Luigi et al., 2020). Furthermore, the more an individual’s time in solitary confinement is prolonged, the more detrimental and harmful the psychological effects become (Haney, 2018). Notably, these findings are akin to many of the results from current research regarding the negative outcomes of the COVID-19 lockdowns.

On the other hand, however, previous research on solitude has found that it can foster creativity, lower self-consciousness, and present the opportunity for self-reflection and emotional renewal. Subsequently, positive solitude results in the cultivation of perceived psychological growth and a heightened level of well-being (Lay et al., 2019). It has been suggested that, from a broader social perspective, the positive benefits of solitude may far outweigh its detriments (Storr, 1988). However, with much of the research on solitude focusing on outcomes of psychological dysfunction, this perhaps promotes the notion that one is more likely to have a negative experience in solitude than a positive one.

On the surface, positive solitude can bolster psychological well-being through empowering one with the freedom to select one’s mental and physical activities without being subjected to inhibiting social burdens (Long & Averill, 2003; Nguyen et al., 2019). Positive freedom of choice and autonomy, coupled with negative freedom from the typical obligations of everyday life, are a combination that has been found to reduce stress and facilitate psychological well-being through elevating feelings of authenticity and of a greater life purpose. Succinctly, these two core elements are what define experiences of distinctly “positive” solitude (Lay et al., 2019; Mor et al., 2020; Nguyen et al., 2018). Taking this understanding further, the self-transformational qualities of positive solitude may be uncovered. Through the removal of oneself from the larger social environment that typically contextualizes and defines identity, solitude offers the opportunity for reconceptualization of the self through the loosening, deconstruction, and ultimately, restructuring of cognitive structures (Long & Averill, 2003). By active, meaningful engagement with one’s thoughts and feelings through the processes of purposeful introspection and self-chosen pursuits, the “self-enhancing” functions of solitude become accessible, therefore nurturing higher levels of self-acceptance, personal growth, and psychological well-being (Thomas & Azmitia, 2019).

The unique conditions of solitude for facilitating exploration of the self intrinsically links it with processes of formation and maintenance of the self-concept, in this case, self-concept clarity. A fundamental, structural aspect of the self-concept, self-concept clarity encompasses clear and consistent beliefs about oneself and one’s place within the larger social environment (Campbell, 1990). A stronger consistency within the self-concept is believed to directly improve emotion regulation (Parise et al., 2019), enrich feelings of authenticity (Chen, 2019), enhance self-esteem (Lee-Flynn et al., 2011), imbue one with a higher perception of life meaning (Błażek & Besta, 2012), and bolster well-being (Lynch & Sheldon, 2017). Opportunities to understand oneself to a greater and more intimate extent, as offered through the medium of solitude, again contributes to and encourages a higher level of psychological well-being (Kraus et al., 2011; Usborne & Taylor, 2010).

A propensity for solitude is considered to develop during adolescence, wherein the discovery of autonomy through the freedom of choice to spend time alone, and to choose how one engages with that time, results in positive experiences of solitude (Corsano et al., 2006; R. W. Larson, 1997). And for older adults, solitude is experienced in a more positive way for those securely ensconced within worthwhile relationships with family members and friends (Hill et al., 2022; Pauly et al., 2018). A conspicuous group to have been neglected from meaningful research on experiences of positive solitude is that of young adults: in current context, this being the millennial generation. Raised during the technology boom of the millennium, millennials are a generation defined by the digital landscape (Buzza, 2017). Living in a state of ever-connectedness where cooperation and collaboration are considered the sacred key to success (Cain, 2012; Kilian et al., 2012), this group of individuals has been raised and treated as part of a collective for their entire lives, beginning with the rise of “helicopter parenting” (Odenweller et al., 2014) and continuing with the global application of conceptualizations of social learning throughout all levels of academia and workplace alike (Gallegos & Peeters, 2011; Johnson & Johnson, 2009; Zuber-Skerritt, 2002). Even configurations of open-plan office spaces has entrapped employees in inescapable connection (Baldry & Barnes, 2012; Laughton & Thatcher, 2019). The importance of the group and of collaborative, extroverted behavior has been deeply instilled within the subconscious of the millennial generation, resulting in a subset of individuals who have experienced life almost as anything but individual. So, what may experiences of solitude be like for millennials?

## Study Rationale and Aims

Considering that young adulthood is posited as being a significant life stage wherein one is challenged with curating many different context-specific self-representations into a coherent self-identity (Diehl & Hay, 2010), it becomes a state of interest as to how millennials, as the current generation of young adults undertaking this task of organizing their identity, have coped with the sudden and dramatic change to their social landscape. Millennials are a cohort who place a high value on leisure time (Buzza, 2017; Mihelič & Aleksić, 2017), suggesting a tendency to seek opportunities wherein they can remove themselves from burdensome, cultural connection, and embrace an element of autonomy through freedom of choice. Therefore, it could be surmised that the sudden sequestration from the larger social environment, as determined by the mitigating measures defined by the government imposed national lockdowns, holds the potential to have been a relatively positive experience for some millennials. The lockdowns may have unexpectedly granted millennials the high value time they desire. For those millennials who reported having a positive experience of the lockdowns, what was this experience of solitude like? Furthermore, how could this have affected their perceived self-growth, nurturance of the self-concept, and ultimately, their psychological well-being? Guided by the literature, the intent of this study was to explore millennial experiences of positive solitude during the U.K. COVID-19 lockdowns. Despite the adverse circumstances in which the state of solitude had been imposed upon this group of individuals, the opportunity for psychological benefits to have arisen from it deserves due attention.

## Method

### Participants

Although no specific generational boundaries are defined, millennials are broadly categorized as those born between the early 1980s and the late 1990s. For the parameters of this study, the working boundaries defining millennials were set to people born between the years of 1981 and 1996 (Dimock, 2019). Accordingly, individuals between the ages of 25 and 40 years were invited for the study.

For the purpose of this study, the chosen working definition of solitude, as directed by the literature, is the disengagement from the demands of the larger social and physical landscape. Therefore, those living with partners or a family unit, or indeed sharing their dwelling with any other individuals, were included in this study. People who continued working through the lockdown were excluded from the study, as were people who transitioned to work from home. Their digital connection to their colleagues and work environment continued to present them with a significant interaction with a large part of their usual social environment, albeit by non-traditional means. Therefore, they were excluded from experiencing solitude beyond the physical definition. All of the participants indicated that they experienced a prolonged time in solitude during the COVID-19 U.K. lockdowns for the duration of at least 2 months, and all identified their experiences as being somewhat positive.

Participants were recruited via convenience sampling through word-of-mouth or public, digital adverts placed on the social media platforms Instagram and Facebook. There was no incentive or compensation offered for participation within the study. Eleven participants expressed interest in the study. One participant was excluded due to geographical location, and two did not respond to further information; their potential participation was not pursued any further. Eight participants in total were included in the study. They were between the ages of 25 and 35; three defined themselves as male, four as female, and one as transgender. Full participant descriptive details can be found in Table 1.

**Table 1.** Participant Demographics.

Name	Age	Gender	Pronouns	Race / ethnicity	Nationality	Time in solitude (months)	Occupation
Ash	26	Trans	They / he	White / Mixed	British	6	Theater designer
Eric	33	Male	He / him	White	British	14	Bar & venue owner
Hannah	31	Female	She / her	White	British	3	Interior designer / farmer florist
Kate	31	Female	She / her	White	British	4	Graphic designer / illustrator
Lucy	32	Female	She / her	White	British	11	Wedding stationer
Luke	32	Male	He / him	White	British	2	Trainee video game programmer / Museum venue manager
Marcus	31	Male	He / him	White	British	10	Guitar teacher
Roisin	25	Female	She / they	White / Irish	Irish	4	MA student / bookseller / journalist

*Note*. All names have been changed to pseudonyms.

### Procedure

For the purpose of the study, an in-depth, semi-structured interview schedule was designed. This format was chosen to foster naturally flowing conversation between the researcher and participant in an endeavor to gain a rich and authentic data set. The full interview schedule consisted of seven core questions, each including various prompts and follow-up questions to help the participant parse the questions in a way that would encourage them to speak openly and at length. The questions included “Do you think your understanding of solitude has changed from what it was before the lockdowns?” and “Has your experience of solitude changed the way you think and feel about yourself?” exploring the participants’ experience of solitude during the lockdowns. Although inclusion within the study required participants to have already identified their experiences of solitude as being somewhat positive, the interview schedule was purposefully designed with neutrality so as to capture the experiences of the participants to the fullest extent and with authenticity. Interviews were conducted within the months of April and May 2021. As per the government-imposed restrictions regarding social mixing at the point of interview, these were conducted online via the video conferencing software Microsoft Teams (MS Teams). MS Teams caters for ease of access as it does not require the participant to download any specific software onto their home device. In addition, conducting the interviews online allowed the participant the opportunity to take part in the interview from the comfort of their own home, further fostering the natural flow of conversation.

The study was designed and carried out in line with the British Psychological Society’s Code of Ethical Practice, and full ethical approval was obtained from the university’s School of Social Sciences Ethics Committee before research commenced. Preceding the interviews, each participant was emailed an information sheet detailing the study in full and a consent form which they had to sign and return by email. Once consent had been obtained, the participant was offered a set of dates and times for potential interview from which they had to select one. Once the interview time was agreed upon, the researcher set up the meeting on MS Teams and the interview could proceed. At the beginning of the interview itself, the participant was verbally reminded of their right to terminate the interview at any point and of their right to withdraw their data in the weeks following. At the end of the interview, the participant was given a verbal debrief, in which they were asked to take a few moments to sit with any thoughts or emotions that might have risen during the course of the interview, to ensure they were comfortable and happy to continue their day with little disruption. A digital copy of the debrief, which additionally contained contacts for appropriate mental health organizations (should they have felt the need for any further support in the aftermath of the interview), was also emailed to each participant.

Eight interviews were conducted in total. Once eight interviews were complete, it was agreed upon by both researchers that data collection had reached saturation and therefore no further interviews were conducted. The average length of the interviews was approximately 47 min, with the shortest interview lasting 34 min and the longest 1 h 6 min. All interviews were recorded using the in-built recording software on MS Teams, with both visual and audio recording captured. The interviews were then manually transcribed, in full, by the researcher/interviewer.

Due to the nature of the study, Interpretative Phenomenological Analysis (IPA) was chosen as the most appropriate form of data analysis. This particular style of thematic analysis allows the researchers to envelop themselves within the data, thus gaining a unique understanding of the personal, lived experiences of the individual participants. In turn, the intimate insights that are gleaned from the individual data can be used to form a more detailed and holistic understanding of the intricate nuances of the phenomenological experience (Smith, 2017) of positive solitude, with which all participants identified.

Both authors were deeply involved in the data analysis. While the project was led by the first author, the second author, with prior experience within qualitative research and IPA methodology, worked closely alongside to offer expert guidance and to nurture the full potential of the research. The process of analysis was lengthy and in-depth, first requiring each researcher to individually and diligently read, and re-read, the transcripts to become familiar with the texts. Once metabolized, the researchers independently annotated the transcripts with interpretive notes of interest before coding each in detail, thus facilitating the emergence of common themes among the data. The researchers then re-joined to discuss their individual analyses and check for cohesion between their results, upon which the set of prominent themes was agreed. Analysis was drawn close to the data to ensure an honest and authentic interpretation of the lived experiences that the participants generously presented within the study.

## Results

Analysis of the data, from all eight participants, revealed how the millennials in the study have closely nurtured and upheld their psychological well-being throughout the unexpected adversity of the U.K. COVID-19 national lockdowns. The conditions, offered by solitude, granted the participants the unique opportunity to consolidate the many different roles within their individual lives into more coherent self-concepts. This process of consolidation was inherently aided by the ability to freely engage in positive freedom of choice in combination with negative freedom from social and societal demands. Under analysis, the data revealed how these freedoms assisted in creating the environment and opportunity for positive transformations to each participant’s sense of self and consequently for buoying psychological well-being.

Three prominent themes were identified, together forming a compelling narrative of self-transformation through the discovery, maintenance, and solidifying of the self-concept, as aided by the unique conditions of positive solitude. The first superordinate theme of *coming to a self-discovery* explores the incongruence that the participants experienced between their socially active lives and their personal beliefs and desires. Continuing within this theme are explorations of positive and negative freedoms of solitude and what impact these freedoms have had on each participant’s sense of self. The second superordinate theme follows the participants as they find ways of *cultivating a more congruent self*. Identified by meaningful engagement in cognitive processes, this superordinate theme offers insight as to how the participants explored and bolstered their developing schematic identities, and how engagement with worthwhile solitude aided this process. To conclude, in *perceived self-transformation*, the outcomes of having developed a more coherent self-concept can be seen, as the participants embrace new kindness toward themselves and display a more positive psychological well-being. Superordinate and subordinate themes are illustrated in Table 2.

**Table 2.** Superordinate Themes and Subordinate Themes.

Superordinate themes	Subordinate themes
Coming to a self-discovery	Inauthenticity of the self in a social world
	Space to breathe
	Finding autonomy through play
Cultivating a more congruent self	Authenticity through mindful activity
	Enhancement through meaningful reflection
Perceived self-transformation	Compassionate liberation
	A strengthened sense of self

### Coming to a Self-Discovery

The unexpected opportunity of solitude during COVID-19 appears to have offered the participants a chance of self-discovery. Through their removal from the typical social environment, the participants were able to take a step back, view, and acknowledge the incongruence they were experiencing through the necessity of playing various context-specific roles that felt inauthentic. The first subordinate theme introduces the initiation of awareness of this incongruence for the participants, as through discussion they brought to light their experiences of *inauthenticity of the self in a social world*. The following two subordinate themes illustrate how the situational solitude of the lockdowns offered opportunity for the cultivation of a stronger self-concept through a greater alignment of inward cognitive attitudes and beliefs with outward experiences and behaviors, as presented by the allied domains of positive solitude: positive and negative freedom. Negative freedom from social demands granted the participants *space to breathe*, while positive freedom of choice offered the participants the opportunity of *finding autonomy through play.*

### Inauthenticity of the Self in a Social World

As the participants were offered a chance of reflection, many discussed the discomfort they experienced as they performed to the demands of context specific social roles. Eric remarked on the frustration of inauthenticity through how he simply cannot “act how he feels” within his customer facing role:

Yeah, it’s just like . . . it’s the customer sort of facing roles. You know, it’s the same with any, like, retail and any sort of hospitality, I suppose. It can just . . . when you’re having a bad day but you can’t just be like . . . *blurgh!* and you can’t just like, actually *act* how you feel. It’s a bit like . . . *grits teeth* . . . yeah. It can be frustrating at times, but . . . *tut* . . . you know . . . (Eric)

The tension and discomfort Eric often felt within this role, as he held back his authentic emotions for the purpose of presenting with the public, is palpable in his sentiment. He may accept the roles that are required of him, but the malaise is abundantly clear. The following extract from Hannah echoes in sentiment, though her attitude is more direct and knowing toward the discomfort her public, work role causes her to feel:

I barely thought about them. It’s really bad! I didn’t miss them; I wasn’t sorry not to be there. I was absolutely loving the fact that I didn’t have to go to *** and I could do my thing. I didn’t miss people. (Hannah)

### Space to Breathe

Negative freedom from the demands of the larger social environment was spoken about liberally by all participants. Regularly expressed as being able to “breathe,” this suggests that negative freedom can be felt not only in the metaphorical, psychological space of having a clearer mind, but also in a more tangibly visceral sense through the physical relaxation of the body. Eric illustrates this through the relief he experienced when granted time away from the constant performance and responsibility required of working closely with an inebriated public:

But, you know, running the bar . . . *ooft* . . . It just, it kinda like, just saps all of your energy. You know? It’s kinda like, faces constantly and, like, the alcohol element and like having to sorta be responsible for people can be . . . urgh, it’s kinda exhausting. So, uh, yeah. It’s been, it’s been kinda a relief, to be honest, for a bit. [. . .] It’s been, it’s been a bit of a breather. (Eric)

Ash talks of the performance of their frantic and highly social working life and how, through the opportunity presented by the lockdown, they realized that they were potentially headed down a dangerous path toward burnout. They mention a reattribution to where they place value, showing appreciation for the opportunity to slow down, breathe, and notice:

At the start, I was very reluctant, because I’d kinda worked myself really hard, literally just before, uhm, everything . . . kinda . . . closed. So, I was still in that headspace of like Work! Work! Work! But then, afterwards, I think I started to realize that I’d maybe overworked myself? And actually, having that time to kinda like, sit and work out where I was placing, like, value on stuff that I was doing was . . . was actually very . . . very helpful. (Ash)

For Lucy, the freedom from the demands of navigating her children through a highly social and energized schedule was abundantly clear. Her life in lockdown radiated relief and enjoyment, as the family were able to slow down and reclaim their time and themselves:

FREE. Totally free . . . ! Yeah! It was like, in terms of day-to-day life, we’d wake up early, go outside and have breakfast in the garden. You’re not looking at the clock, you’ve not got to be there—you’ve not got to be anywhere! So, there’s no, there was no school run to do, no gymnastics clubs to go to, it doesn’t really matter what day of the week it is. (Lucy)

### Finding Autonomy Through Play

All participants engaged thoroughly with Positive freedom. Speaking about their choices of personal endeavor with genuine sincerity and joy, they chose to fill their time with a variety of conspicuously creative activities. Lucy reflected on her experience within the context of her whole family unit, as they all relished the opportunity to entertain whatever sparked their imaginations:

The whole family was just creating constantly. Yeah, we all tried our hand at different forms of art—in the garden, inside, uhm, music . . . yeah, it was just a really creative, free time. (Lucy)

Five of the participants already had a defined propensity for creativity but had previously found themselves stifled by having to focus their creative energy on work-related projects. Ash talked about the opportunity to repurpose the act of drawing for personal enjoyment, speaking about this with pleasant simplicity:

I think then also I started to . . . feel creative again. In a, in a really positive way. Like, I thought, I suddenly realized like—Oh, I actually, I, you know, I do, I draw for my job but, I also really *like* drawing. I started drawing because I really like drawing. And that was just something that I actually hadn’t done in a really long time. Is just sat and drew for myself. And it’s something that I have continued, actually, as well, which is . . . really nice—haha. (Ash)

Hannah’s experience somewhat reflects that of Ash. Here, she expresses the pleasure in having the opportunity to simply “play” with her floristry, purely for her own enjoyment:

I did loads of, actually, loads of experiments with like, floristry, which was nice. So, I just got to play, actually! I get really creative with making flowers for *nobody* but me. (Hannah)

### Cultivating a More Congruent Self

The next undertaking for the participants was that of cultivation and conservation of the cognitive schemas that they were developing through new engagements with both positive and negative freedoms. *Cultivating a more congruent self* was exhibited in two distinct ways. The first was the art of finding a*uthenticity through mindful activity*, wherein participants mindfully engaged with practical pursuits as a means to create the mental space necessary for cognitive processes to occur. The second was *enhancement through meaningful reflection*, whereby participants embraced the opportunity of solitude to allow for moments of quiet introspection.

### Authenticity Through Mindful Activity

Creative and tactile activities became a way of mindful process for many of the participants. Regarding playing his guitar, simply for the pleasure of it, Eric delightfully referred to this joyful act as “noisy meditating,” alluding to the mindful aspects of being fully absorbed in the authentic moment and lost in the creative process:

I suppose it’s like—it’s almost like meditating! In a way, I suppose. It’s just really noisy meditating—haha! (Eric)

Kate discussed how when she was engaged with creative pursuits she was able to allow her mind to think about and process things in a comfortable way. In addition, she brought to the fore the physical relaxation she experienced while she was immersed in these activities:

[. . .] ’Cause when I’m, just when I’m focused, just doing a drawing or whatever—painting—I just . . . I mean, yeah, my mind is still thinking about stuff but, uhm, my body’s relaxed. (Kate)

Roisin beautifully illustrated the process of finding authenticity through engaging in mindful activity in sharing her experience of being able to “think more productively” by means of gardening:

[. . .] through, like . . . doing something as involved through my hands as, like, planting all these seeds but my head was still, like, I knew that I was doing something and that I had to do this process of, like, I had to go out and water them every day or else they might die, or like, I have to like, plant these shoots into a bigger box or else they wouldn’t grow but, and I knew that, but it didn’t require any more thinking than that. So, then I was able to . . . I don’t know—haha, I don’t know where this is going. I was able to think a bit more about . . . I think I was able to think more productively about, like, things that I had been . . . anxious about, or worrying about, maybe? because I already had something to concentrate on. (Roisin)

A sentiment similarly echoed by Eric; addressing the process more directly, he explained how the physical and practical outlet of energy created a space for him to process his thoughts:

I suppose it’s because you’ve got something to, like, put your . . . your thoughts into. ‘Cause if you’re, I mean . . . yeah, I think if you don’t sorta have some sort of outlet, be it music, or painting, or whatever, what do you do? What do you do with your thoughts and your . . . limbs? (Eric)

### Enhancement Through Meaningful Reflection

Some participants voiced direct thoughts regarding the specifics of reflection for them, and how the natural, though at times uncomfortable, process of self-reflection was an integral part of their perceived psychological growth. Marcus acknowledged the specific need for solitude he required for mental processing and how having the opportunity to actively attend to his thoughts “without being beholden to” others was a significant element of reflective practice for him:

[. . .] purely from a very literal point of view, being on my own and . . . basically being alone with my thoughts . . . allows to, to have this sort of internal, uhm . . . I’m not sure if “internal dialogue” is the right word but, just, sort of, you know, it, it just allows me to manage and run though situations and . . . and deal with things on my own without being beholden to somebody else. (Marcus)

Roisin eloquently referred to the gentle, albeit sometimes uncomfortable, nature of self-reflection as “self-confrontation”:

I guess solitude, part of it requires, like, self-confrontation . . . in some way. And that doesn’t always have to be . . . I dunno, like, a fraught confrontation, it can, like, be quite a gentle thing. (Roisin)

This is echoed in the following extract from Luke, wherein he described the necessity for reflection as a part of personal growth, irrespective of whether it was a completely comfortable process or not, for it “helps you work towards something more positive”:

[. . .] having that opportunity to, kinda, step back from everything and . . . Introspect and . . . You know . . . There are times when that doesn’t necessarily feel completely positive, but I think you can learn a lot out of it that helps you work towards something more positive. (Luke)

### Perceived Self-Transformation

As the participants began to experience an alignment of their inner cognitive beliefs and outward experiences and behaviors, the consolidation of the self-concept became evident. Participants openly discussed *compassionate liberation*, wherein they were able to offer themselves a new level of kindness. Finally, the participants exhibited a *strengthened sense of self* as they embraced a more authentic and congruent self-concept.

### Compassionate Liberation

The participants’ experiences throughout the lockdowns seem to have all been somewhat transformative. A suggested outcome of the developmental journey they have been on during their time in solitude is that of self-compassion. Marcus discussed how he had learnt to offer himself more kindness and lower the value he holds on what he perceived others thought of him:

[. . .] it’s basically allowed me to be a *lot* kinder to myself in terms of the pressures that I put on myself. Uhm, and *allow* me to, to give—to care less about other people’s sort of arbitrary perceptions of me when I’m aware that I’m doing everything that I can to, to be as uhm, you know, motivated and, and successful as I can be. Uhm, and, and by proxy of me . . . *caring* less about, you know, being conscious *less* about what other people may or may not think about me, it’s also made me more conscious of the way *I* feel about me. (Marcus)

Ash similarly offered insight into how their experience had changed their self-perceptions in a positive way, displaying kindness to themselves through metaphorically “patting [themselves] on the back.” Though their quote, as if spoken directly to themselves, shows that they may still need some direct affirmation to solidify this self-compassion and accept its validity. While first steps of perceived growth had been taken, acceptance of their new understanding of themselves in a more compassionate light was still in development:

[. . .] my outlook on that now has definitely changed to something a lot more positive. And kinda patting myself on the back a little bit more and being like “no, you’ve done this and this is GOOD. You’re doing WELL.” (Ash)

Considering herself in solitude along with her family unit, Lucy revealed that through self-reflection she had been able to become more compassionately attuned to herself, but also to her family members, as they also had to each other: growing in compassionate strength not just singularly, but as a close family unit:

I guess solitude in *our* sense of a family unit being on our own means that we are more attuned to each other and ourselves . . . Yeah . . . Listening to our own thoughts rather than relying on people that aren’t us to help us . . . Yeah . . . So, it’s made us a stronger family unit, one hundred percent. (Lucy)

### A Strengthened Sense of Self

Through the participants’ personal developments in solitude, the process, for some, had almost come full circle. Reflecting upon the second subordinate theme of finding *space to breathe*, many participants concluded that their experiences had been somewhat restorative, by regaining a sense of autonomy and a rediscovery of personal identity through facilitation of negative freedom. Ash, reflecting on their highly socialized life before lockdown, revealed their newly re-discovered autonomy in solitude as simply “finding space to be me”:

Just giving myself, like, time to breathe almost. I think that’s what I was missing beforehand. It’s that I do really like socializing, I do really like being around people. Uhm, I didn’t realize that I wasn’t really giving myself just space . . . to . . . be . . . *me.* (Ash)

Having now been exposed to a prolonged time in solitude, Hannah reflected on her understanding of the construct through the lens of her personal experience. Her conceptualization can be seen to change in real time, as she processed her new understanding of what solitude is and what it meant for her:

The first thing that popped into my head was that solitude almost feels like a sad thing. If someone’s on their own, that’s not, uhm, I don’t know . . . It just . . . I’m not sure how to describe it, it’s just how it popped into my head. But then when I think about like . . . I guess “alone time” as a phrase, rather than “solitude,” that makes me feel, like, quite comfortable. It feels quite comfortable because I’m, I can be quite introverted so, uhm . . . I think it’s almost like, yeah, something that’s quite secure, and, because there’s nobody else around it’s quite a safe space. (Hannah)

Finally, Lucy stated her newfound awareness in autonomy and authenticity with joy and enthusiasm. She explicitly highlighted her cognitive transformation and expressed enjoyment in that realization:

I don’t know who I was before this!—haha—you know? And now, I think I do! I’m like . . . Yeah . . . I am a person—haha! (Lucy)

## Discussion

The study aimed to explore how some U.K. young adults experienced positive solitude during the COVID-19 lockdowns. The findings demonstrated that there is a narrative of self-transformation in each participant’s experience. This transformation began with engagement in domains of positive solitude wherein they could explore their personal desires and begin to regain a healthy level of autonomy. As the participants started to experience a more authentic alignment of their inward beliefs and outward behaviors, they then proceeded to undertake the process of cultivating a more congruent sense of self through mindful activity. Finally, the participants acknowledged a perceived self-transformation in which they experienced themselves in a more authentic manner.

To begin their process of self-transformation, the participants had to first identify the incongruence of the self they were experiencing through meeting the expectations of the various social roles within their lives. Through the performance of context-specific selves to meet the necessary demands of workplace personas, participants struggled to understand themselves in an authentic manner, and felt this incongruence innately: “you can’t just like, actually act how you feel,” states one participant through gritted teeth. The incongruence that the participants alluded to can be identified as self-concept differentiation. Here, the individual experiences themselves with different characteristics as determined by the demands of varying social roles, often to the detriment of psychological well-being (Donahue et al., 1993). This division of the self, at the behest of external forces, can breed feelings of inauthenticity (Wood et al., 2008): an experience with a tendency to promote psychological maladjustment (Pilarska, 2016). Potential risks of living with a fragmented self-concept include emotional distress (G. M. Larson & Sbarra, 2015), interpersonal and intrapersonal problems (Pilarska, 2016), and anxiety and depression (Schwartz et al., 2011). It is therefore imperative that a more congruent self-concept can be individually cultivated to mitigate psychological maladjustment and encourage better psychological well-being.

As the participants entered the lockdowns and began to first experience solitude, the sense of relief they expressed, regarding their removal from the larger social environment, was evident. An integral element to the experience of positive solitude is that of negative freedom from the demands of the larger social landscape (Koch, 1994). The relief that negative freedom offers from the burdens of social performance within everyday life is considered a facilitator of psychological well-being (Lay et al., 2019; Mor et al., 2020). In this case, negative freedom had allowed the participants the chance to gain new awareness over themselves by taking both a physical and metaphorical step back from the performative demands required of the larger social environment: a chance to feel “totally free.”

Interacting closely with the concept of negative freedom is the sister element of positive solitude: positive freedom of choice (Long & Averill, 2003). Now unbeholden to the social demands of contextual connection, participants were able to indulge in playful and creative activities. Through gaining the opportunity to engage in desirable behaviors and activities, under their own volition, they were able to reclaim a feeling of autonomy: imperative for positive affective experiences of solitude (Nguyen et al., 2018). The cumulative experiences from all participants regarding their enjoyment of both positive and negative freedoms is indicative of the alignment between outward behaviors and inward cognitive schemas. The participants embraced the opportunity that had arisen, and subsequently embraced a more authentic way of living.

Through acknowledgment of self-concept differentiation, and engagement with both positive and negative freedoms, the participants initiated the organismic, cognitive process of schematic development (Beals, 1998). To cultivate and conserve this development, the participants continually engaged with mindful activities such as gardening, drawing, or playing a musical instrument. In line with previous research suggesting that mindful awareness facilitates clearer representations of the self (Diehl & Hay, 2011), participants detailed that the physicality of the act, and their natural ability to take part in it without any strenuous mental effort, created space for them to comfortably sit with and process thoughts in a productive way. The cognitive process in action here can be explained by working memory theory, wherein finite working memory resources are divided between the task at hand and active thoughts. Therefore, an environment is forged in which thoughts can be processed in a productive manner, rather than allowing them to become overwhelming or problematic (Baddeley, 1992). Whether gardening, playing guitar, or making art, these somatic moments of sustained attention created a calming effect upon the mind. Buddhist teachings consider mindfulness as a means to learning, by creating a sense of continuity and gaining greater wisdom through intentional action, rather than relief from confrontational thoughts with distraction. Subsequently, purposeful engagement with the present is considered to nurture a more authentic life (Brazier, 2013). This classical, Buddhist teaching reflects the cultivation of a more congruent sense of self. Acts of mindfulness encourage insight into the true nature of the self (Vago & David, 2012) resulting in a heightened sense of authenticity: an integral component of self-concept clarity (Chen, 2019).

Rather than engaging in mindfulness through activity, some participants chose to take the opportunity to practice deep thinking and purposeful reflection. It has been suggested that the human capacity for solitude relies upon the ability to engage in deep introspection: reflecting upon and interpreting one’s thoughts and experiences (Lay et al., 2019; Long & Averill, 2003). Although a noble practice, and one with considerable rewards, taking this route was more challenging for the participants, with one reflecting on this process as “self-confrontation.” This mirrors previous research of “self-enhancing” cognitive functions having the potential of being somewhat psychologically painful and uncomfortable (Thomas & Azmitia, 2019). The deliberate act of purposeful reflection suggests a further development and maintenance of the self-concept. Through having the available time to fully attend to one’s thoughts, emotions, and experiences in a meaningful manner, these can then be introduced into schematic development and thus aide in imbuing oneself with a more coherent self-concept clarity (Chen, 2019) and subsequently further adding to feelings of authenticity.

The participants, having undergone the uniquely self-transformative experience of positive solitude, appeared emboldened with a new awareness of their own thoughts and autonomy. Consequently, they had found the ability to offer themselves kindness and compassion. A significant indicator of the journey they had taken, self-compassion is indicative of psychological well-being (Hall et al., 2013) and is linked with higher levels of happiness and optimism and lower levels of depression, anxiety, and negative rumination (Neff, 2009). Through gaining an awareness of themselves and partaking in reflective, mindful activities, the participants had experienced a positive effect on their well-being, in turn allowing themselves to bestow kindness upon themselves in a meaningful and positive way. The newly established levels of authenticity and psychological well-being show a greater level of coherence and consistency within the self-concept (Chen, 2019; Kraus et al., 2011). The transformational properties of solitude were certainly felt and embraced by the participants, as demonstrated by their kinder attitudes toward themselves and a newfound sense of authenticity.

### Reflections and Suggestions for Future Research

It is worth noting that there are several further contributing factors that may have added to the parameters as to why the participants in this study had such success in engaging with positive solitude. The first is one unique to the conditions of the U.K. COVID-19 lockdowns, in that financial government support somewhat limited financial stress for the participants involved in this study. Six of the participants were on furlough for the duration of their time in lockdown, while two participants received government financial aid at a later date. A second condition, and one largely unique to the millennial generation as it currently stands, is a limited caregiving burden. At the point of study, millennials are categorized as between the ages of 25 and 40 (Dimock, 2019), therefore, most do not yet have to care for elderly parents nor do many have children of their own (only one participant in the study had young children to care for). These conditions are worth noting as they will undoubtedly be contributing factors as to how the participants in the study, and perhaps millennials in general, were able to experience positive solitude throughout the course of the COVID-19 lockdowns.

Participants were largely gathered by means of convenience, and therefore some were previously known to the researcher, or were known to each other. Accordingly, this may account for some similarities in experiences, particularly regarding any propensity for creativity.

While homogeneity is key for understanding experience within high-quality qualitative studies (Larkin et al., 2006; Smith, 2017), the current study was discernibly White. Further investigations should focus on experiences of Black and other ethnic minority groups. The LGBTQ+ community was in part represented within the study, but a focus surrounding the LGBTQ+ community in isolation would be worthy of further investigation. Finally, the participants involved were all able-bodied. Therefore, the experience of disabled and neurodivergent individuals should also be studied in isolation. The endeavor of this study was to add to a body of research forming a more holistic understanding of experiences in solitude. To continue that understanding, other specific groups should rightly be given a voice.

### Conclusion

The participants have been shown to achieve better psychological well-being through the lockdowns by embracing the uniquely self-transformative qualities that positive solitude offers. Initially, negative freedom from the demands of the larger social environment alleviated the self-concept differentiation the participants typically experienced. No longer did they have to alter their outward characteristics to meet the burdensome requirements of performing the varying social roles within their lives. The negative freedom that the lockdowns established created a physical and psychological space to breathe for the participants. This granted relief from feelings of inauthenticity and allowed for the amelioration of stress upon both the mind and body. In turn, the participants were then able to engage in positive freedom, wherein they could exercise autonomy through engaging in playful activities of their own choosing and under their own volition. Through seizing the opportunity to participate in continued mindful activity and meaningful reflection, the participants were able to cultivate a more congruent self-concept. In doing so, this achieved an alignment of the inward cognitive schemas of the self and outward behaviors and characteristics. Subsequently, participants were imbued with a new sense of authenticity, the ability to offer themselves more compassion, and heightened levels of psychological well-being. Much as R. W. Larson (1990) originally proposed regarding those with a propensity for solitude, the participants were able to deftly turn the terrifying situation of the COVID-19 lockdowns into a pleasant one, through embracing solitude as a journey toward finding themselves (Storr, 1988).

This study highlights the value of positive solitude in promoting feelings of authenticity, elevating psychological well-being, and decreasing stress and depression related symptomology. For solitude to be experienced as positive, this study reveals that it is down to how the solitude is engaged with by the individual, rather than any physical or practical conditions under which it is delivered. Through seeking positive solitude, the opportunity to freely engage in playful activities is created. Within this, an individual can embrace momentary feelings of authenticity by taking part in activities that align with their inward beliefs and values. Alternatively, through mindfulness, an individual can create an environment to address troublesome thoughts in a productive manner. By having the opportunity to engage in these solitude-related activities in earnest, a more congruent self-concept can be cultivated, and the individual can benefit from heightened feelings of authenticity, be able to express more kindness toward themselves, and experience elevated psychological well-being. Consequently, stress- and depression-related symptomology become reduced. Clinical implications for the study concern the importance of mindfulness and other solitude-promoting interventions as a psychotherapeutic method for the promotion of authenticity and psychological well-being, and for the amelioration of negative rumination, stress, and depressive symptomology (Segal et al., 2002). The intention of this study is to add to a small, but growing, body of work surrounding the benefits of solitude. Furthermore, it is to offer validation for those who have had an alternative experience within the U.K. COVID-19 lockdowns to the otherwise predominantly reported negative outcomes.
